# Neurosurgical Outcomes in Severe Traumatic Brain Injuries Between Service Lines: Review of a Single Institution Database

**DOI:** 10.7759/cureus.37445

**Published:** 2023-04-11

**Authors:** Maxwell A Marino, Imran Siddiqi, Lana Maniakhina, Patrick M Burton, Louis Reier, Jason Duong, Dan E Miulli

**Affiliations:** 1 Neurosurgery, Riverside University Health System Medical Center, Moreno Valley, USA; 2 Medical School, Edward Via College of Osteopathic Medicine, Spartanburg, USA; 3 Medical School, Cleveland Clinic Indian River Medical Center, Vero Beach, USA; 4 Neurosurgery, Desert Regional Medical Center, Palm Springs, USA; 5 Neurosurgery, Arrowhead Regional Medical Center, Colton, USA

**Keywords:** modified rankin scale(mrs), gcs (glasgow coma score ), injury severity scores (iss), brain trauma injury, institutional outcome, neurosurgery nursing, neuro-critical care, surgical intensive care, icp monitoring, post traumatic brain injury

## Abstract

Severe traumatic injury (sTBI) continues to be a common source of morbidity and mortality. While there have been several advances in understanding the pathophysiology of this injury, the clinical outcome has remained grim. These trauma patients often require multidisciplinary care and are admitted to a surgical service line, depending on hospital policy. A retrospective chart review spanning 2019-2022 was completed using the electronic health record of the neurosurgery service. We identified 140 patients with a Glasgow Coma Scale (GCS) of eight or less, ages 18-99, who were admitted to a level-one trauma center in Southern California. Seventy patients were admitted under the neurosurgery service, while the other half were admitted to the surgical intensive care unit (SICU) service after initial assessment in the emergency department by both services to evaluate for multisystem injury. Between both groups, the injury severity scores that evaluated patients' overall injuries were not significantly different. The results demonstrate a significant difference in GCS change, modified Rankin Scale (mRS) change, and Glasgow Outcome Scale (GOS) change between the two groups. Furthermore, the mortality rate differed between neurosurgical care and other service care by 27% and 51%, respectively, despite similar Injury Severity Scores (ISS) (p=0.0026). Therefore, this data demonstrates that a well-trained neurosurgeon with critical care experience can safely manage a severe traumatic brain injury patient with an isolated head injury as a primary service while in the intensive care unit. Since injury severity scores did not differ between these two service lines, we further theorize that this is likely due to a deep understanding of the nuances of neurosurgical pathophysiology and Brain Trauma Foundation (BTF) guidelines.

## Introduction

Severe traumatic brain injury (sTBI) is a significant source of morbidity and mortality worldwide [1]. It occurs when external physical forces induce a change in brain function or anatomy, including, but not limited to, diffuse axonal injury and intracranial hemorrhage. In the US, traumatic brain injury (TBI) is most commonly caused by unintentional falls, motor vehicle crashes, suicide, or homicide [1]. Though our understanding of the pathophysiology of traumatic brain injury is now extensive, this understanding has not translated to a related improvement in outcomes [2]. According to the Centers for Disease Control (CDC), over 70% of patients with sTBI deteriorate or never improve clinically. Furthermore, direct and indirect medical fees amount to approximately $76.5 billion per year [3]. Therefore, the associated lifetime financial costs and health-related problems pose an emotional and economic burden on the family and the healthcare system.

Prognostication factors determining outcomes in TBI patients have been forthcoming in the literature, including biomarkers [4]. Predicting clinical outcomes plays a significant role in patient management and resource allocation. Since 1974, the Glasgow Coma Scale (GCS) has served as a powerful measure of injury and has been found to be a prognosticator of clinical course [5]. It assesses the level of consciousness by analyzing eye, verbal, and motor responses. The GCS stratifies patients with TBI into three categories: mild, moderate, and severe. Patients with a GCS of eight or less fall under the severe category and carry a worse prognosis [6]. However, it is important to note confounding variables that may obscure the scoring system, such as incomplete resuscitation, seizures, drug intoxication, intubation, and sedation [7].

At our hospital, an isolated head and spine injury will be admitted to the neurosurgery service. Given that TBI commonly occurs in the setting of other injuries, including long-bone fractures and soft tissue injuries, those multi-system injured patients are admitted to various trauma services depending on hospital policy and clearance by the trauma service. Taking these factors into consideration, severe TBI has become a pathology treated in a multidisciplinary setting [8].

According to the American Association of Neurological Surgeons (AANS) and Congress of Neurosurgeons (CNS)'s Position Statement on Neurosurgeons and Neurocritical Care 03.03.09, "Neurosurgeons are fully trained in neurointensive care by reason of training program requirements, and upon completion of training are competent to independently manage and direct treatment of patients with neurological disorders requiring critical care" [9].

For neurosurgical trainees, ensuring appropriate management and exposure to severe TBI patients is critical. The Accreditation Council of Graduate Medical Education (ACGME) defines neurological surgery as a medical discipline and surgical specialty that provides care for adult and pediatric patients in the treatment of pain or pathological processes that may modify the function or activity of the central nervous system (e.g., brain, hypophysis, and spinal cord), the peripheral nervous system, (e.g., cranial, spinal, and peripheral nerves), the autonomic nervous system, and the supporting structures of these systems (e.g., meninges, skull and skull base, and vertebral column) and their vascular supply (e.g., intracranial, extracranial, and spinal vasculature) [10]. Treatment encompasses non-operative management (including prevention, diagnosis, image interpretation, and neurocritical intensive care and rehabilitation) and operative management (including image interpretation, endovascular surgery, functional and restorative surgery, stereotactic radiosurgery, spinal fusion, and instrumentation). Thus, to adequately train a neurosurgeon, they must be appropriately exposed to the intensive care management of a severe TBI patient, and attending neurosurgeons must be well-versed and trained in the appropriate management of these patients.

Over time, a neurosurgeon’s role has evolved to include the prevention of neurotrauma, the development of trauma protocols, providing acute neurosurgical and neurocritical care, and making transfer decisions [11].

Trauma patients with sTBI are managed according to the Brain Trauma Foundation's (BTF) evidence-based guidelines in order to prevent secondary injury and optimize outcomes [12]. The recommendations outline when to utilize hyperosmolar therapy, seizure prophylaxis, deep vein thrombosis (DVT) prophylaxis, mechanical ventilation, intracranial pressure (ICP) monitoring, jugular bulb monitoring, cerebral perfusion pressure (CPP) threshold, blood pressure threshold, and nutrition. Moreover, the BTF guidelines provide indications for decompressive craniectomy and cerebrospinal fluid (CSF) drainage, which are both part of the neurosurgeon’s armamentarium [7].

Neurosurgeons have a responsibility to provide the highest level of care, including prompt diagnosis and management of the consequences of neurological impairment. Oftentimes, the severity of a neurological injury is underrecognized, especially in the elderly, who may have an altered physiology. Furthermore, TBI patients may develop a "frontal lobe paradox", which leads to impaired cognitive, emotional, and behavioral problems rather than physical symptomology [13]. As a result, without prompt neurosurgical evaluation and continued evaluation, subsequent follow-up and care are in jeopardy [11].

The goal of using standardized treatment in sTBI is to reduce the risk of developing secondary brain injury. Patients with sTBI are prone to developing cerebral edema, anoxic brain injury, hemorrhage, and ischemia if not promptly treated and properly monitored for these complications [14]. We hypothesize that TBI patients admitted to a neurosurgery service can be safely managed by a well-trained neurosurgeon with intensive care unit experience, and there may be improved outcomes when allowing for other injuries compared to those admitted to other service lines for patients with isolated severe traumatic brain injury, secondary to a deeper understanding of neurophysiology and the neurosurgical nuances of BTF guidelines. Therefore, it is emphasized that implementing neurosurgeons’ input at an earlier stage of sTBI diagnosis is critical to the outcome when working with a multidisciplinary team specially trained in TBI.

## Materials and methods

A retrospective analysis of the Arrowhead Regional Medical Center (ARMC) Neurosurgery Census was performed. This is a level-one trauma center located in Colton, Southern California. This study was approved by the Arrowhead Regional Medical Center Institutional Review Board (approval 20-26). The waiver of informed consent was approved by the IRB. Upon approval by the IRB, 140 consecutive adult patients from 2019-2021, ages 18-99, with an admission diagnosis of severe traumatic brain injury (GCS 3-8) after resuscitation by the trauma or neurosurgery teams, were abstracted from the electronic medical record system. Demographic parameters of age, sex, and race were recorded. Due to an electronic medical record system switch, the number of consecutive patients was limited to 70 in each category.

These patients were further stratified according to several parameters, including admission service, intensive care unit (ICU) length of stay (LOS), total hospital LOS, neurosurgical intervention, GCS score on admission and discharge, Glasgow Outcome Scale (GOS) on discharge, modified Rankin Scale (mRS) on discharge, presence of an ICP monitor, Injury Severity Scale (ISS), disposition, and in-hospital mortality.

The Glasgow Coma Scale (GCS) is a neurological scale used to assess the conscious state of a person following a traumatic brain injury. The GCS is determined by assessing the patient's response to verbal commands, eye opening, and motor responses. The GCS ranges from a score of three to 15, with higher scores indicating a better level of consciousness. The Glasgow Outcome Scale (GOS) is a scale used to measure the long-term outcome following a traumatic brain injury. It consists of five categories: death, vegetative state, severe disability, moderate disability, and good recovery. The GOS is often used to compare different treatments and evaluate the effectiveness of a particular intervention.

The modified Rankin Scale (mRS) is a scale used to measure the degree of disability or dependence in the daily activities of a person who has suffered a stroke or other disabling illness or injury. It is one of the most widely used scales in the field of neuroscience, as it provides a standardized way to measure a patient's outcome in terms of their ability to take care of themselves and participate in daily activities. The mRS consists of six categories, ranging from no symptoms (score 0) to complete dependence (score 5). Category six includes patients who are dead, while category five includes patients who are alive but are completely dependent on others for all activities of daily living. The mRS is an important tool for assessing the long-term outcome of a stroke or other disabling condition.

The Injury Severity Score (ISS) is a tool used in the field of trauma medicine to assess the severity of injuries sustained by a patient. This score is based on the Abbreviated Injury Scale (AIS), which is a tool used to describe and rate the severity of individual injuries. The AIS assigns a score to each injury, with one being the least severe and six being the most severe. The ISS ranges from one to 75, with higher scores indicating more severe injuries. A score of one indicates a minor injury, while a score of 75 indicates that the patient’s injuries are so severe that they are likely to be fatal.

Two groups were studied. Group one included patients admitted to the neurosurgery service, while group two included patients admitted to the surgical intensive care unit (SICU) service. Patients were admitted to the neurosurgery service after evaluation by the trauma service and the neurosurgery service. Patients with multisystem injuries were typically admitted to the SICU service unless the neurosurgical disease process was so severe but not catastrophic that it was determined after discussion that the patient should be admitted to the neurosurgery service. Similarly, if patients with a minimal multisystem injury were non-operative, the surgical ICU service would defer admission to the neurosurgery service and provide consultation-based services. Patients admitted to either service would be admitted to the same physical ICU units with the same nursing staff. The attending surgeon of the primary service, whether SICU, orthopedic, or neurosurgeon, retained responsibility while the trauma patient was under their care, requiring that they be informed of and concur with major therapeutic and management decisions. A student’s t-test was utilized to compare the quantitative variables, including ICU LOS, total hospital LOS, GCS on admission and discharge, GOS, ISS, and mRS, between the two groups. Pearson’s chi-square test was used for the analysis of categorical variables such as neurosurgical intervention, mortality, and disposition between groups. For proportions, a Z-test for differences in proportions was used between groups. A p-value less than or equal to 0.05 was deemed statistically significant. Findings are expressed as means for quantitative data.

## Results

Reviewing the single institution census, 140 consecutive patients were identified as demonstrating severe TBI. Out of this group, 70 were admitted to the neurosurgery (NSX) service, while the remaining patients were admitted to the surgical intensive care unit (SICU) trauma service. Trauma patients requiring ICU admission must be admitted to a surgical service. We measured outcomes as the changes in the Glasgow Coma Scale (GCS), Glasgow Outcome Scale (GOS), and modified Rankin Scale (mRS) from admission to discharge for all patients.

The presentations of both groups are depicted in Table 1. Patients in the neurosurgery service were slightly older, with an average age of 48.5 years, compared to those in SICU, with a mean age of 42.6 years (p=0.035). The assigned sex between the groups was comparable without a statistical difference. The average GCS on admission varied by one point between neurosurgery service (GCS 6) and SICU service (GCS 5), which was deemed statistically significant. Lastly, the initial modified Rankin Scale and Glasgow Outcome Scale were similar between the two groups without any statistically significant difference. The mean ISS score, when considering all patients, was 41.4 for the neurosurgery group and 41.9 for the SICU service (p=0.44). However, for patients with ISS scores 51-75, the means were significantly different; 70 for the neurosurgery group compared to 75 for the SICU service. The SICU team clearly had more of the more severely traumatized patients. For patients with an ISS of 0-50, the mean ISS for the neurosurgery service was 31.56, compared to 34.4, which was not a statistically significant difference.

**Table 1:** Demographics and severity of TBI *: statistically significant; (SD): standard deviation

	Neurosurgery service n=70	SICU service n=70	p-value
Age (years), mean (SD)	48.5 (20.2)	42.6 (19.4)	0.035*
Sex, n	17W, 53M	15W, 55M	0.072
GCS on admission, mean (SD)	6 (2)	5 (2)	0.021*
mRS on admission, mean (SD)	4.53 (0.50)	4.41 (0.50)	0.089
GOS on admission, mean (SD)	2.90 (1.40)	2.31 (1.54)	0.098
ISS score 0-75, mean	41.44	41.91	0.44
ISS score 0-50, mean	31.56	34.37	0.093
ISS score 51-75, mean	70	75	0.013*

The results depicted in Table 2 demonstrate a difference in ICP monitor utilization with neurosurgery service using it on 65/70 (92.86%) of patients, whereas when admitted to SICU, it was implemented by the neurosurgeon on 48/70 (68.57%) of patients (p<0.001). In our local practice, an ICP monitor is always an external ventricular drain placement. To explain this, it was notable that of those five patients who did not receive an ICP monitor on the neurosurgery service, four were deemed catastrophically injured beyond intervention, and their injuries resulted in death. The other single patient that did not receive an ICP monitor was a special case in custody where attaining consent versus emergency placement was deliberated; however, the patient improved rapidly enough that no ICP monitor was then needed; the patient was discharged to an acute rehabilitation center. The patients in SICU who did not receive ICP monitoring include 16 of the 17 patients with an injury severity score of 75. The other five patients consisted of those with an ISS above 50 and a single patient with an ISS of 48. None of these 22 patients were offered ICP monitoring as their injuries were catastrophic and intervention, including ICP monitoring, was deemed futile as evidenced by no single patient in this group surviving.

**Table 2:** Length of stay (LOS) and ICP monitoring *: statistically significant; (SD): standard deviation

	Neurosurgery service n=70	SICU service n=70	p-value
ICP monitor utilization	92.85%	65.87%	0.00064*
ICU LOS (days), mean (SD)	17.57 (19.16)	7.77 (7.75)	0.000006*
Hospitalization LOS (days), mean (SD)	23.60 (22.50)	14.06 (16.17)	0.001*

Additionally, there was also a significant difference in the length of hospitalization and ICU stay. sTBI patients on neurosurgery service experienced longer treatment in the ICU (mean 17.6 days) and longer hospitalization (average 23.6 days). More neurosurgical patients were sent to a long-term acute care facility (LTAC) and rehab, 16/70 (23%) and 4/70 (6%), respectively. Alternatively, there was no statistically significant difference in acute rehabilitation unit (ARU), home, skilled nursing facility (SNF), or transfer disposition between the two groups.

After analyzing three different neurological scales, patients on the neurosurgery service with sTBI achieved an improved outcome that was statistically significant (Table 3). On average, the GCS score on discharge significantly improved by 3.7 points in patients on the neurosurgery service compared to the other group with a mean of 2.26 (p=0.029). Similarly, neurosurgical patients improved by 0.63 points on average according to the modified Rankin Scale, whereas the other surgical service patients deteriorated by 0.11 points (p=0.02). This finding is supported by a similar outcome in GOS improvement. Patients under the neurosurgery service had an improvement in GOS scores, with a mean increase of 0.23 points. In contrast, patients under the other surgical service had a decrease in GOS points on average of 0.20 (p=0.031). For patients with an ISS of 0-50, the GCS score was significantly improved by 3.9 points in patients on neurosurgery services compared to the SICU group with a mean of 2.33 (p=0.038). Patients under neurosurgery service had an increase in GOS scores with a mean change of 0.25 points. In contrast, patients under the SICU service had a decrease in GOS points on average of 0.175 (p=0.05). Neurosurgical patients trended to improve by 0.61 points on average according to the modified Rankin Scale, whereas SICU patients improved by 0.05 (p=0.08).

**Table 3:** Patient outcomes based on service provision *: statistically significant

	Neurosurgery service n=70	SICU service n=70	p-value
GCS change	+3.70	+2.26	0.029*
mRS change	-0.63	+0.11	0.020*
GOS change	+0.23	-0.20	0.031*

**Figure 1:**
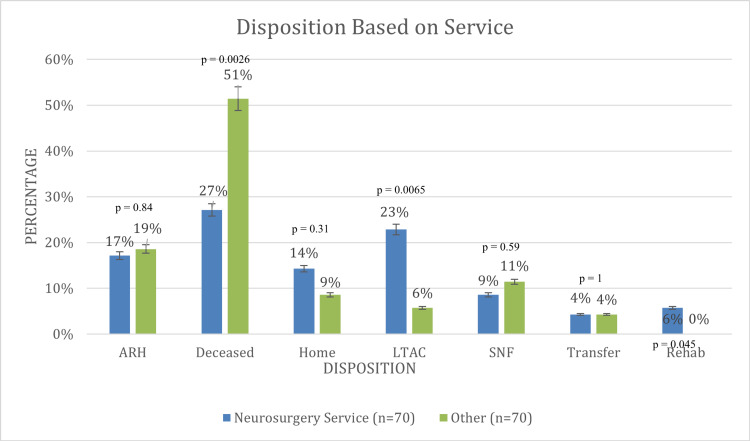
Disposition based on service provision ARH: acute rehab hospital; SNF: skilled nursing facility; LTAC: long-term acute Care

The disposition of sTBI patients based on surgical service varied between the groups (Figure 1). Of note, 19/70 (27%) patients died under the neurosurgical service, and 36/70 (51%) patients died under the SICU service (p=0.0026), once again demonstrating the effects of the most severe multi-system injury on head injuries. Of the 19 patients who died on the neurosurgery service, three were in the ISS strata of 16-25, eight were in the ISS strata of 26-50, and eight were in the ISS strata of 51-75. All patients on the neurosurgery service with ISS 51-75 had a mortality rate of 100%. Accordingly, on the SICU service, of the 36 patients who died, two were in the ISS strata of 16-25, 13 were in the ISS strata of 26-50, and 21 were in the ISS strata of 51-75 (91% of SICU patients in the ISS 51-75 strata resulted in mortality). The differences in the percent mortality of sTBI patients between neurosurgery and SICU in their respective ISS strata were not statistically different.

A subgroup analysis was done that included the ISS 0-50 from both the neurosurgery service and SICU service. The mean ISS was found to be 31.6 and 34.4 for the neurosurgery and SICU trauma services, respectively, and there were not statistically significant differences between groups. However, when considering the ISS from 51-75, the mean ISS of 75 from the SICU trauma service was found to be statistically greater than the mean of 70 on the neurosurgery service. Considering the population of patients in the ISS range of 0-50, a subsequent analysis of outcomes was done, including changes in GCS, GOS, and mRS, summarized in Tables 4, 5.

**Table 4:** Patient outcomes based on service provision, injury severity score of 0–50 * = statistically significant

	Neurosurgery service n=70	SICU service n=70	p-value
GCS change	+3.90	+2.33	0.038*
mRS change	-0.61	-0.05	0.080
GOS change	+0.25	-0.175	0.05*

**Table 5:** Patient mortality based on service provision, stratified by ISS

	Neurosurgery service			SICU service			
ISS	Total n	n= Death	% Death	Total n	n= Death	% Death	p-value
16-25	36	3	8.33%	6	2	33.33%	0.08
26-50	26	8	30.77%	41	13	31.71%	0.936
51-75	8	8	100.00%	23	21	91.30%	0.389

## Discussion

Patients with sTBI have extremely high mortality and morbidity rates and often have severe multi-system injuries [1]. The care of these patients requires challenging medical and ethical decision-making. Thus, critically ill patients benefit from a multidisciplinary approach. Although multiple services are involved, one of the surgical services takes the lead and makes the final decision in the sTBI patient’s care. The primary team must rapidly recognize and promptly treat acutely life-threatening conditions.

The results demonstrate improved clinical outcomes in neurosurgical patients with isolated sTBI who are primarily on the neurosurgery service when they do not have significant systemic trauma.

Severe TBI patients demonstrate good recovery, as witnessed by improved GOS and mRS scores from admission to discharge. We theorize that this is likely due to a deep understanding of the nuances of neurosurgical brain trauma guidelines, strict neurological monitoring, and prompt care due to recognized differences in neurophysiology from general systemic physiology in terms of fluid, blood pressure, ICP, ventilator, sedation, seizure, edema, nutrition, DVT prophylaxis, and other management. In a neurological intensive care unit, patients undergo daily frequent detailed serial neurological assessments, including GCS, cranial nerve, and spinal cord exams. Attention to subtle neurological changes may prompt the neurosurgeon’s rapid, definitive treatment of life-threatening conditions such as elevations of intracranial pressure. Subsequently, the neurosurgical team’s ultimate decisions may streamline treatment, including decompressive craniectomy or EVD placement.

When considering the assignment of trauma patients to surgical service lines, it is important to consider the overall injury severity and systemic injury burden of the patient. Based on the outcomes described, the Injury Severity Score (ISS) may be an important tool to guide this decision-making process for sTBI patients. The ISS allows healthcare professionals to assess the severity of a patient’s injuries quickly and accurately [15]. This information is critical in determining the course of treatment needed and in predicting the patient's outcome. For example, a patient with an ISS of one is likely to recover quickly and without complications, while a patient with an ISS of 50 is likely to require extensive medical and surgical intervention and may have a worse prognosis. This information can be used to develop better treatments and interventions for trauma patients with severe injuries. When comparing the cohort of patients with sTBI, the SICU service line did indeed have a worse mean ISS, but when accounting for the effects of a multi-system injury on a severe head injury and using the ISS of 50 as a cutoff, the mean ISS between service lines was not significantly different. Among these patients with an ISS of 50 or less, the GCS score was improved by 3.9 points in patients on the neurosurgery service compared to the other group with a mean of 2.33 (p=0.038). Patients under neurosurgery service had an increase in GOS scores with a mean change of 0.25 points. In contrast, patients on the SICU service had a decrease in GOS points on average of 0.175 (p=0.05). The SICU service had a higher proportion of patients in the most severe ISS category (51-75), with a mortality rate of > 90%, resulting in greater mortality among that service line’s patients as well. Therefore, in patients with an ISS of 50 or less in the setting of isolated severe TBI, the neurosurgery service may be more appropriate for primary admission and therefore ultimate decision-making. However, there is a need for SICU management with neurosurgical consultation in the most severely injured sTBI patients, those with an ISS greater than 50. However, there are some limitations to the Injury Severity Score, which is based solely on the severity of injuries. For example, it does not consider the location of injuries or the patient's age, sex, or medical history.

The sTBI patient with an isolated brain injury can be best served by admission to the neurosurgery service, with assistance from other trauma surgeon specialists, in a neurointensive care unit (NeuroICU), which is a specialized unit within a hospital that is dedicated to the care of critically ill patients with neurological conditions [11]. Patients admitted to the neuroICU with sTBI are staffed by healthcare professionals who are highly trained and experienced in the management of neurological conditions’ trauma. These neurotrauma specialists, including neurosurgeons, have a deep understanding of the non-operative and surgical nuances of the nervous system, which allows them to provide optimal care for patients with neurological conditions that are often slightly different from other traumatologists and intensivists [11]. Possible differences in management have been identified but are not limited to prompt procedural intervention, fluid selection (both crystalloid and colloid), sodium management, oxygenation, blood pressure parameters, seizure recognition, and sedation.

The neuroICU and SICU are equipped with advanced monitoring technologies that allow the neurosurgeon to closely monitor patients' neurological status [13]. These technologies can detect changes in a patient's condition and help healthcare professionals intervene quickly, recognizing the dependency of the central nervous system on each part of the body to better optimize outcomes.

Patients in a neuroICU under the primary care of the neurosurgeon with close assistance from other traumatologists and intensivists are constantly watched by nurses trained in the pathophysiology of the nervous system who will prompt intervention and management of complications that can arise from neurological conditions, such as seizures, brain swelling, or hemorrhages [11]. This dedicated nursing staff can improve patient outcomes and reduce the risk of long-term neurological damage by being keenly aware of these acute neurological changes that may portend larger, catastrophic changes.

NeuroICU and SICU patients receive interdisciplinary care, which means healthcare professionals from different specialties work together to provide comprehensive care to prevent primary and secondary injury to the central nervous system [11]. This includes emotional, psychological, spiritual, and social support. In addition to the neurosurgeons, there are other trauma specialists, critical care specialists, neurologists, critical care nurses, physical therapists, social workers, pharmacists, clergy, and others. This team-based approach can help ensure that patients receive comprehensive care all the time and help families navigate the complex and often stressful experience of caring for a loved one with a severe traumatic brain injury.

There are limitations to a study at a single level-one trauma institution that analyzes outcomes of patients with severe traumatic brain injury admitted to different service teams. The study may not be generalizable to other hospitals or populations. The study may be biased if the patients who were admitted to different service teams had different severity of the injury or other factors that could affect their outcomes, which was accounted for with ISS stratification; however, a detailed analysis may yet reveal further nuance. The study may not be able to account for all of the factors that could affect the outcomes of patients with traumatic brain injury. Despite these limitations, the study can provide valuable information about the outcomes of patients with traumatic brain injury and can also help identify areas where further research is needed.

## Conclusions

We compared the outcomes of patients with severe TBI between those admitted to neurosurgery and SICU services. Patients under neurosurgery had lower mortality, better cognitive outcomes, and more invasive monitoring. These findings suggest that early neurosurgeon involvement in severe TBI management improves outcomes. Patients with isolated sTBI and ISS 0-50 can be managed primarily by the neurosurgical service with extensive assistance from other services. Patients with sTBI and an ISS greater than 50 should be managed primarily by the SICU service, with extensive assistance from the neurosurgeons and other services. Severe traumatic brain injury is a complex medical condition that requires a multidisciplinary approach and a profound understanding of the nervous system’s pathophysiology to ensure that patients receive timely and appropriate interventions, tailored treatment plans, and support throughout the intensive care phase of treatment in order to obtain the best possible outcomes for patients. Further research is necessary to understand whether additional demographic markers or confounding variables influence the distinctions between service outcomes, as well as whether these improvements can be translated to other services through the creation of further clinical management guidelines.
